# The Differentiation *in vitro* of Human Tonsil B Cells With the Phenotypic and Functional Characteristics of T-bet+ Atypical Memory B Cells in Malaria

**DOI:** 10.3389/fimmu.2019.00852

**Published:** 2019-04-24

**Authors:** Abhijit A. Ambegaonkar, Satoshi Nagata, Susan K. Pierce, Haewon Sohn

**Affiliations:** ^1^Laboratory of Immunogenetics, National Institute of Allergy and Infectious Diseases, National Institutes of Health, Rockville, MD, United States; ^2^Center for Drug Design Research, National Institutes of Biomedical Innovation, Health and Nutrition, Osaka, Japan

**Keywords:** atypical memory B cells, malaria, T-bet, B cell receptor signaling, TLR9, IFN-γ

## Abstract

Malaria is a deadly infectious disease associated with fundamental changes in the composition of the memory B cell (MBC) compartment, most notably a large expansion of T-bet^+^ MBCs, termed atypical MBCs. However, we know little about the precursors of atypical MBCs and the conditions that drive their differentiation. We compared the responses of human tonsil naïve B cells, MBCs, and germinal center B cells to a variety of stimulatory conditions. We determined that prolonged antigen presentation in the presence of CpG and IFN-γ induced maximal expression of T-bet and other phenotypic markers of malaria-associated atypical MBCs primarily in naïve B cells *in vitro*. Importantly T-bet^+^ naïve-derived B cells resembled atypical MBCs in their hypo-responsiveness to signaling through their B cell receptors. Thus, naïve B cells can be induced to differentiate into phenotypically and functionally atypical-like MBCs *in vitro* under conditions that may prevail in chronic infectious diseases *in vivo*.

## Introduction

For many pathogens, an individual's single exposure leads to life-long immunity. This phenomenon, immunological memory, is encoded, in part, in a population of high affinity, long-lived memory B cells (MBCs) that upon re-exposure to the same pathogen are responsible for the production of high affinity, high titer protective antibody responses. However, not all pathogens induce protective immunity and for such pathogens the infection becomes chronic. It is now well-appreciated that many chronic infections are associated with alterations in the composition of the MBC compartment. Indeed, the chronic infections, HIV, malaria, and tuberculosis, are each associated with large expansions in an unusual or atypical population of MBCs (1–4). In malaria, these atypical MBCs share several characteristics of classical MBCs, including similar isotype distributions, replicative histories and IgV gene repertoires (2). However, as we know little about the processes of proliferative expansion and IgV gene usage or somatic mutation during chronic malaria exposure it is not possible to draw conclusions about the relationships of atypical and classical MBCs based on these observations.

Atypical MBCs can be distinguished from classical MBCs by their expression of cell surface proteins and their transcriptional profile (2, 3, 5, 6). Our recent studies provided evidence that relative to classical MBCs (CD19^+^ CD21^+^ CD27^+^), atypical MBCs (CD19^+^ CD21^−^CD27^−^) isolated from peripheral blood of adults with lifelong exposure to malaria differentially upregulated *TBX21* that encodes T-bet, the Th1-lineage defining transcription factor (6). Nearly 80% of atypical MBCs *ex vivo* expressed T-bet by flow cytometry and of these over 60% showed high expression of T-bet that correlated with the expression of additional atypical MBC markers (6). These atypical MBC markers included a variety of surface proteins including CD11c, CD86, CD95, and CXCR3 as well as inhibitory receptors including FcRL5, CD85, CD32B, and CD22, and decreased expression of CD35, CD40, CXCR5, CD62L, and CCR7 (2, 3, 5). Immediately *ex vivo*, atypical MBCs in malaria-exposed individuals have high basal levels of phosphorylated kinases in the B cell receptor for antigen (BCR) signaling pathway as compared to conventional MBCs and upon BCR crosslinking *in vitro* the fold change in phosphoproteins in atypical MBCs is less than that of conventional MBCs (2, 6). Sequencing of V_H_ and V_L_ genes from individuals in malaria-endemic areas revealed the presence of atypical and classical MBCs that encoded broadly neutralizing antibodies against *Plasmodium falciparum*, the parasite that causes malaria (7). *Plasmodium falciparum* specific antibodies were also detected in the serum of these individuals, although direct secretion of these antibodies by atypical MBCs was not shown. Notably, atypical MBCs do not proliferate nor secrete cytokines or antibodies in response to a variety of stimulants *in vitro* and in this regard, appear dysfunctional (2).

Antibodies play a key role in naturally acquired immunity to malaria and yet for children living in malaria endemic areas the process of acquiring protective antibodies is remarkably slow requiring years of repeated infection with *P. falciparum* (8). Malaria immunity is manifest by the ability to resist clinical febrile malaria and individuals in malaria endemic regions only rarely acquire resistance to infection (9). The acquisition of resistance to clinical malaria is accompanied by increases in classical MBCs and long-lived antibodies (10, 11) but also by a large expansion of atypical MBCs (2, 5). Because atypical MBCs do not appear to secrete cytokines or antibodies upon activation, it has been postulated that atypical MBCs may contribute to the inefficient acquisition of malaria immunity (2). However, it is equally possible that atypical MBCs promote the acquisition of resistance to febrile malaria and in their absence the acquisition of malaria immunity would be even less efficient.

The nature of the precursor B cell that differentiate into an atypical MBC during chronic infectious diseases and the mechanisms that drive differentiation are only poorly understood. The common features of human chronic infections including persistent antigen activation and inflammation *in vivo* have been suggested to play roles in the expansion of atypical MBCs. Indeed, we recently showed that T-bet expression was induced in human peripheral blood naïve B cells *in vitro* by exposure to IFN-γ in presence of soluble IgM-specific antibodies to crosslink the BCR (6).

Here we investigated the conditions under which human tonsil B cells were induced to express T-bet and other atypical MBC markers *in vitro*. We choose to evaluate B cells from tonsil tissue as these may better reflect the response of B cells in secondary lymphoid tissues during chronic infections as compared to peripheral blood B cells. We provide evidence that prolonged stimulation of B cells over hours by antigen bound to membranes that mimic presentation by follicular dendritic cells (FDCs) (12) in the presence of the inflammatory cytokine, IFN-γ, and the TLR9 agonist, CpG, induced the majority of naïve B cells to express high levels of T-bet as well as a variety of other surface markers associated with atypical MBCs. To a lesser extent tonsil MBCs were also stimulated under these conditions to differentiate into cells with the characteristics of atypical MBCs. However, germinal center (GC) B cells were relatively unresponsive to these stimulation conditions. Of course, it is possible that under other stimulatory conditions *in vitro* MBCs and GC B cells may be induced to undergo differentiation toward atypical MBC, for example through Tfh cells or CD40. These naïve B cell-derived T-bet^+^ B cells also showed high basal levels of phosphorylated kinases in the BCR signaling pathway and attenuated antigen-induced BCR signaling characteristic of atypical MBCs in malaria. These results suggest that *in vivo* atypical MBCs may be the product of persistent antigen presentation by FDCs to naïve B cells or MBCs combined with TLR activation by parasite products in the highly inflammatory environment that accompanies febrile malaria.

## Results

### Antigen, CpG, and IFN-γ Induce High T-Bet Expression in Human Tonsil B Cells

Given the closest correlation between the high expression of T-bet with atypical MBCs in malaria, the induction of T-bet expression and its magnitude may be an indicator of differentiation toward atypical MBCs. Several lines of evidence showed that antigen, the TLR 9 agonist, CpG, the cytokines IL-12 and IL-18 and IFN-γ are major components of T-bet induction in B cells (6, 13–20). To understand how these stimulants influence the magnitude of T-bet induction and its expression in B cell subsets, we tested the effect of the combination of these components in human tonsil B cells. B cells were purified to >99% by negative selection from human tonsils obtained from several individuals. The composition of purified CD19^+^ tonsil B cells based on CD10 and IgD expression was ~33% naïve B cells (IgD^+^ CD10^−^), 10% MBCs (IgD^−^CD10^−^), and 47% GC B cells (IgD^−^CD10^+^) (Supplementary Figure 1A). B cells were incubated *in vitro* for 40 h under a variety of stimulation conditions implicated in inducing the expression of T-bet in B cells both in humans and in mice *in vivo* and *in vitro*. Variations in the conditions included the form of the antigen provided to the B cells, the cytokine environment (either IFN-γ or IL-12 + IL-18) and the presence or absence of the TLR9 agonists, CpG. As a surrogate antigen we used F(ab′)_2_ goat antibodies specific for human λ and κ chains (referred to here simply as antigen). Antigen was provided either in a soluble form or presented on stiff planar lipid bilayers (PLBs), mimicking FDCs or flexible plasma membrane sheets (PMSs), mimicking DCs (12). Since antigen on PLB cannot be easily internalized by B cells in contrast to antigen on PMS that B cells readily internalize (12), antigen on PLB may be continuously engaged by B cells through their BCRs providing prolonged BCR engagement as compared to antigen on PMS in which engagement is followed by internalization.

**Figure 1:**
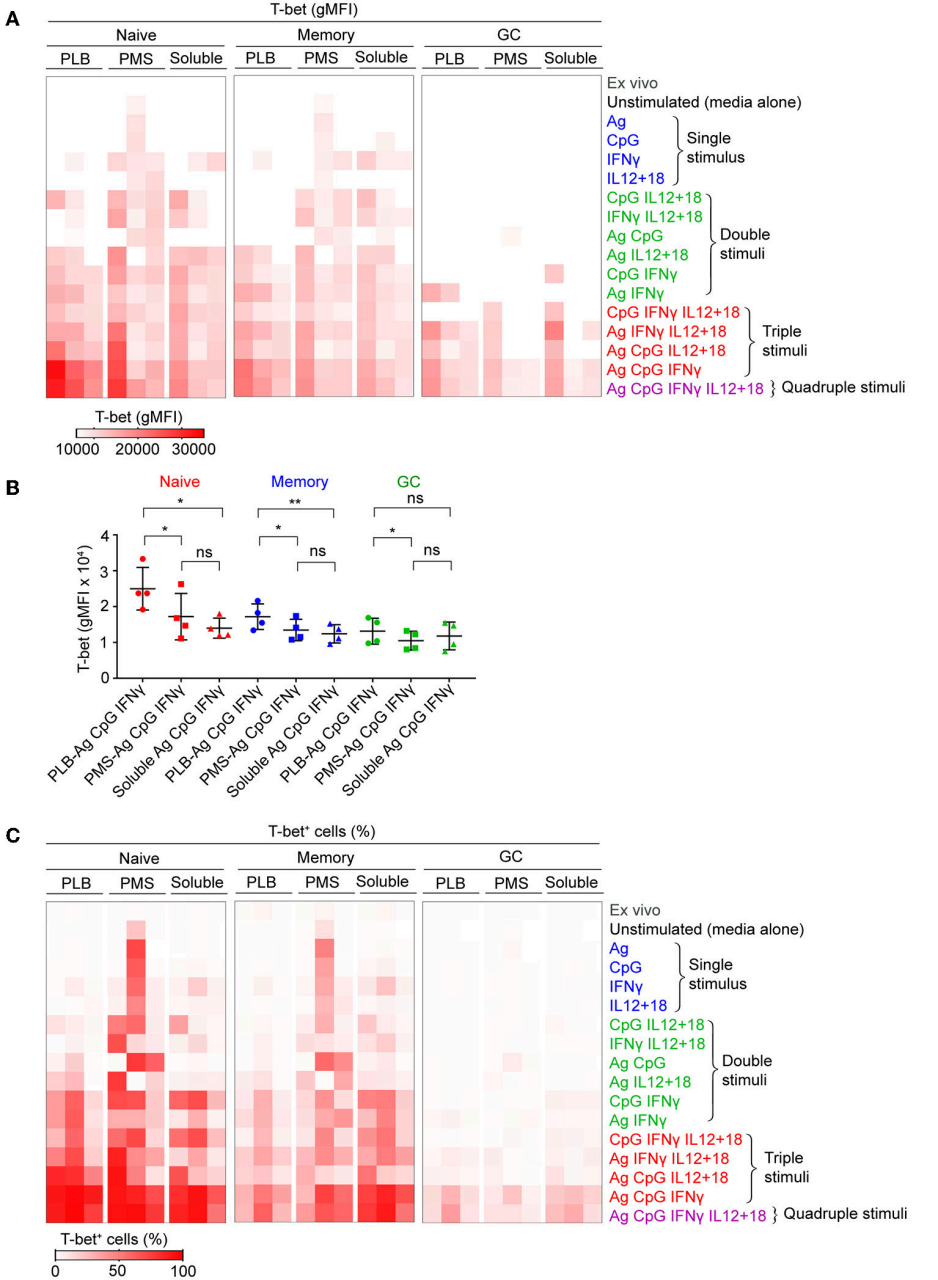
Tonsil B cells express T-bet upon BCR, TLR9, and IFN-γ stimulation *in vitro*. Tonsil B cells were cultured *in vitro* for 40 h with combination of antigen, either soluble or presented on a planar lipid bilayer (PLB) or plasma membrane sheet (PMS), CpG, IFN-γ, or IL-12 + IL-18. T-bet expression in naïve (IgD^+^CD10^−^), memory (IgD^−^CD10^−^), and GC (IgD^−^CD10^+^) B cells was analyzed by flow cytometry. **(A)** Heat map indicating T-bet expression (gMFI) by naïve, memory, and GC B cells of the three individuals (columns) from three representative experiments. The stimulation conditions are as indicated to the right of heat map and are grouped into single, double, triple, and quadruple stimuli. The columns are subgrouped according to the mode of antigen presentation either attached to PLB or PMS or soluble. The gMFI is calculated for conditions in which a minimum of 5% of the cells were T-bet^+^. **(B)** Comparison of T-bet expression (gMFI) by naïve, memory, and GC B cells stimulated *in vitro* with antigen either attached to PLB or PMS or soluble, in the presence of CpG and IFN-γ (*n* = 4). Data for T-bet expression (gMFI) by naïve, memory, and GC B cells stimulated *in vitro* on all stimulation conditions is provided in Supplementary Table 1. Data were analyzed using one-way analysis of variance (ANOVA) with Tukey's adjustment. ^*^P < 0.05; ^**^P < 0.01; ns, not significant. **(C)** Heat map indicating the percent of T-bet^+^ cells for each of the conditions in **(A)**.

We first tested the ability of combinations of antigen on PLB, CpG, and IFN-γ to induce T-bet expression in B cells purified from seven different donors (Supplementary Figure 1C). For all seven individuals we observed highly significant differences in the response of their naïve B cells to the different conditions over a 40 h incubation with a combination of antigen on PLB, CpG, and IFN-γ inducing the highest levels of T-bet expression. We then expanded the combinations of stimuli, incubating purified B cells subpopulations with antigen, CpG, IFN-γ, or IL-12 + IL-18 alone (single stimulus) or in combinations of two (double stimuli) or three (triple stimuli) or with all four (quadruple stimuli) (Figure 1A). B cells were recovered from culture after 40 h and analyzed by flow cytometry for the expression of T-bet, IgD, and CD10 to identify naïve-, MBC- and GC B cell-derived T-bet^+^ B cells. Viability of stimulated and unstimulated B cells ranged between 60 and 70% (Supplementary Figure 1A). Flow cytometry analyses of purified B cells before and after 40 h culture in the absence of stimulation showed that the expression of markers that discriminate naïve, MBCs and GC B cells (IgD and CD10) were relatively stable *in vitro* (Supplementary Figure 1A). T-bet expression was determined by flow cytometry and representative flow cytometry plots for B cells obtained from the tonsils of one representative individual that were either unstimulated or stimulated *in vitro* with combinations of antigen presented on PLB, IFN-γ, CpG, and IL-12 + IL-18 are shown for IgD^+^ CD10^−^ (naïve), IgD^−^ CD10^−^ (MBC), and IgD^−^ CD10^+^ (GC B cell) at the end of the 40 h culture (Supplementary Figure 2). The gMFI of T-bet expression by B cells above that of unstimulated controls in the indicated gates (Supplementary Figure 2) were summarized as a heat map (Figure 1A) and data table (Supplementary Table 1). The highest levels of T-bet expression for the three individuals appeared to be in naïve-derived B cells stimulated on antigen presented on PLB in the presence of CpG and IFN-γ (Figure 1A). The addition of IL-12 + IL-18 to the antigen, CpG and IFN-γ containing cultures appeared to have little effect on T-bet expression. A quantitative comparison of the gMFI of T-bet expression by naïve B cell-, MBC-, and GC B cell-derived B cells obtained from four donors' tonsils including three shown in the heat map confirmed that the highest T-bet expression was by naïve-derived B cells provided with antigen presented by PLB, in the presence of CpG and IFN-γ (Figure 1B).

We also determined the percent of B cells present in the T-bet^+^ gate under each condition and these data were presented as a heat map. Nearly 100% of naïve-derived B cells showed a shift in T-bet expression in response to each of the three forms of antigen in the presence of IFN-γ and CpG (Figure 1C) although the T-bet gMFI was only consistently high among the three individual donors for B cells stimulated with antigen presented on PLB (Figures 1A,B). Large percentages of MBC-derived B cells also shifted into the T-bet^+^ gates (Figure 1C), although the gMFI of cells in these gates was lower than that of naïve-derived B cells (Figure 1A). Very few GC-derived B cells shifted into the T-bet^+^ gates (Figure 1C) and most of these had comparably low T-bet gMFIs (Figures 1A,B).

**Figure 2:**
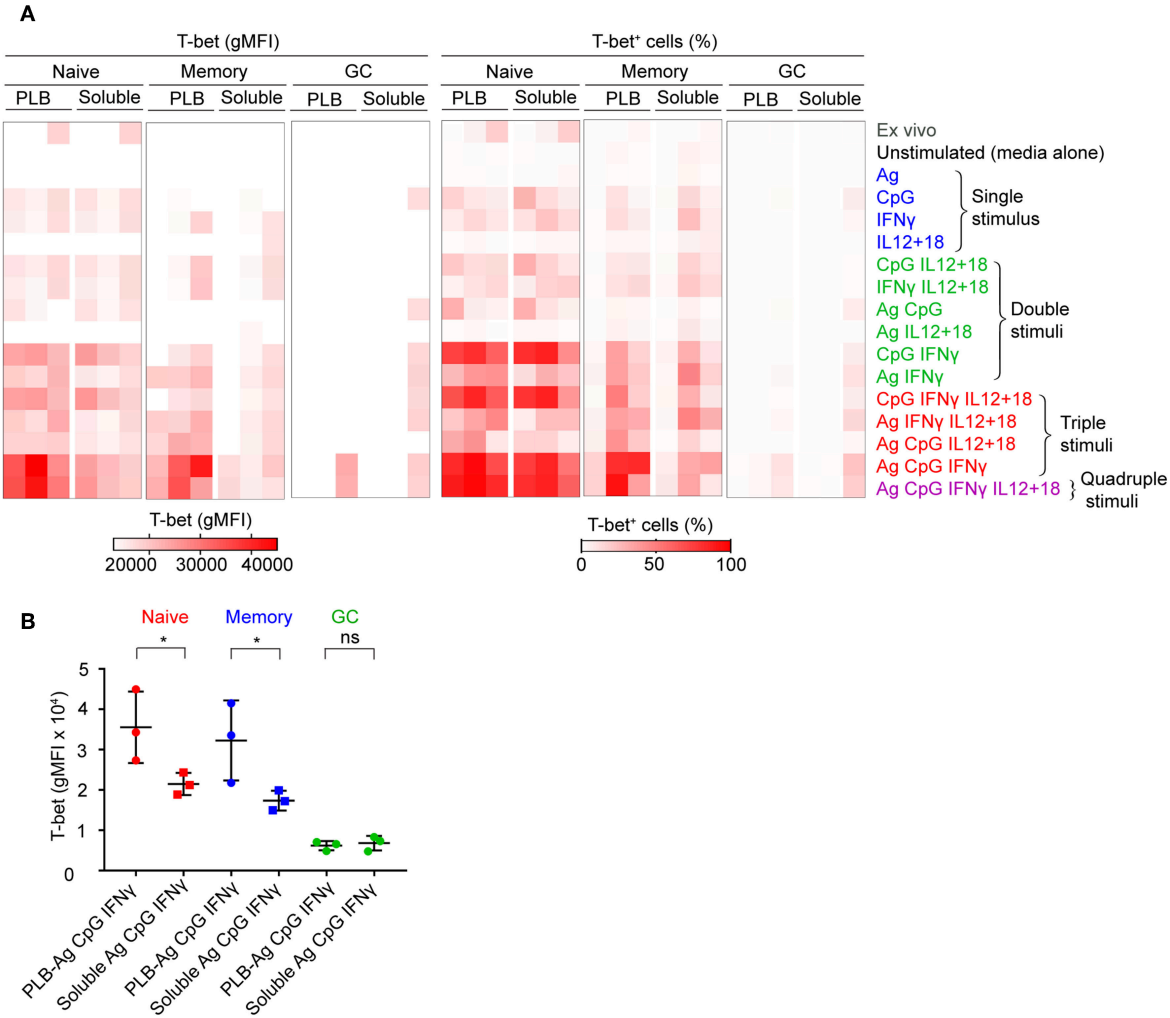
T-bet expression in FACS sorted naïve, memory, and GC B cells upon stimulation *in vitro*. **(A)** Tonsil B cells from three individuals were FACS sorted based on IgD and CD10 expression into naïve (CD10^−^IgD^+^), memory (CD10^−^IgD^−^), and GC (CD10^+^IgD^−^) B cells gated as shown (Figure S1B) and cultured *in vitro* as in Figure 1A with either soluble antigen or antigen presented on PLB in the presence of the stimuli shown. T-bet expression was analyzed by flow cytometry and presented as a heat map indicating T-bet expression (gMFI) (left panel) and the percent of B cells expressing T-bet (right panel). The gMFI was calculated for conditions in which a minimum of 5% of the cells were T-bet^+^. **(B)** Comparison of T-bet expression (gMFI) by FACS sorted naïve, memory, and GC B cells stimulated *in vitro* with antigen either attached to PLB or soluble, in the presence of CpG and IFN-γ (*n* = 3). Data for T-bet expression (gMFI) by FACS sorted naïve, memory, and GC B cells stimulated *in vitro* on all stimulation conditions is provided in Supplementary Table 1. Data were analyzed using paired *t* test. ^*^P < 0.05.

To verify the contributions of naïve, MBCs and GC B cells to T-bet^+^ cells under the conditions described, we sorted tonsil B cells into these three subpopulations gated as shown (Supplementary Figure 1B) before culture *in vitro* with soluble antigen or antigen presented on PLB with various combinations of CpG, IFN-γ, and IL-12 + IL-18. The viability of stimulated and unstimulated sorted B cell populations after 40 h in culture ranged between 40 and 70% (Supplementary Figure 1B). The gMFI of T-bet expression was quantified as were the percent of B cells within the T-bet^+^ gate as in Figure 1. The results were comparable to those of unsorted cells (Figures 2A,B, Supplementary Figure 3), verifying that naïve human tonsil B cells stimulated with antigen presented on PLB, in the presence of IFN-γ and CpG gave rise to B cells expressing high levels of T-bet. Given that unsorted human tonsil B cells and sorted populations gave similar results in terms of T-bet expression, we carried out further characterization of T-bet^+^ cells using unsorted purified human tonsil B cells stimulated *in vitro*.

Taken together these data show that the highest T-bet expression can be induced in naïve B cells and a subset of MBCs placed on antigen presented on PLBs, mimicking FDC, in the presence of the TLR9 agonist CpG and the inflammatory cytokine, IFN-γ.

### T-Bet^+^ B Cells Express an Array of Markers Characteristic of Malaria-Associated Atypical MBCs

**Figure 3:**
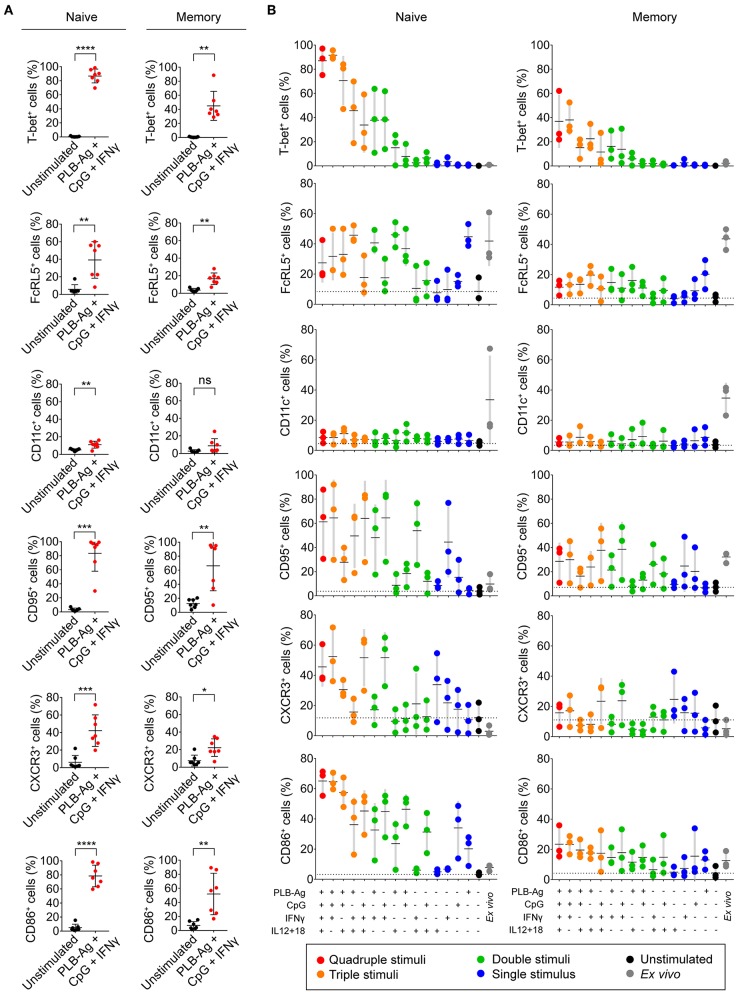
Tonsil naïve and memory B cells upregulate malaria-associated atypical MBC markers upon BCR, TLR9, and IFN-γ stimulation *in vitro*. **(A)** Tonsil B cells were cultured *in vitro* for 40 h unstimulated or stimulated with antigen presented on PLB in the presence of CpG and IFN-γ. Comparison of the expression of T-bet, FcRL5, CD11c, CD95, CXCR3, and CD86 determined by flow cytometry for naïve (IgD^+^CD10^−^) and memory (IgD^−^CD10^−^) B cells are given as the percent of B cells expressing each marker (*n* = 7). Data were analyzed using paired *t* test. ^*^P < 0.05; ^**^P < 0.01; ^***^P < 0.001; ^****^P < 0.0001; ns, not significant. **(B)** Percentage of naïve and memory B cells expressing T-bet, FcRL5, CD11c, CD95, CXCR3, or CD86 either immediately *ex vivo* or after stimulation *in vitro* for 40 h under the conditions indicated. Each symbol represents a single individual (*n* = 3). Black bars indicate the mean value and gray boxes indicate ± 1 s.d. Dotted line indicates mean value for unstimulated cells.

**Figure 4:**
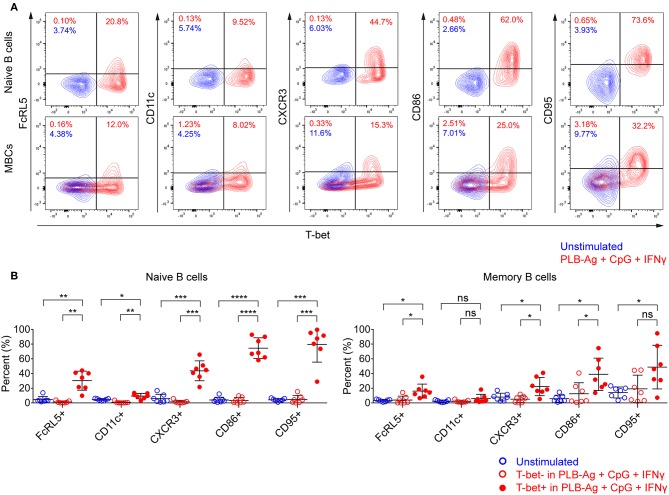
T-bet^+^ cells induced *in vitro* also express malaria-associated atypical MBC markers. Tonsil B cells were cultured *in vitro* for 40 h unstimulated or stimulated with antigen on PLB in the presence of CpG and IFN-γ. naïve (IgD^+^CD10^−^) and memory (IgD^−^CD10^−^) B cells were analyzed by flow cytometry for the expression of T-bet and FcRL5, CD11c, CXCR3, CD86, and CD95. **(A)** Representative flow cytometry plots indicating expression of FcRL5, CD11c, CXCR3, CD86, or CD95 by T-bet^+^ naïve and memory B cells. **(B)** Comparison of expression of FcRL5, CD11c, CXCR3, CD86, or CD95 by unstimulated or stimulated T-bet^+^ and T-bet^−^ naïve and memory B cells (*n* = 7). Data for expression of these surface markers by T-bet^+^ naïve and memory B cells stimulated *in vitro* on all the stimulation conditions is provided in Supplementary Table 2. Data were analyzed using one-way analysis of variance (ANOVA) with Tukey's adjustment. ^*^P < 0.05; ^**^P < 0.01; ^***^P < 0.001; ^****^P < 0.0001; ns, not significant.

In addition to T-bet, atypical MBCs associated with malaria express elevated levels of several cell surface markers including FcRL5, CD11c, CD95, CXCR3, and CD86 (2, 5, 6). We determined the percent of naïve- and MBC-derived B cells that were induced to express each of these markers when cultured with antigen presented on PLB in the presence of CpG and IFN-γ. Tonsil B cells from a total of seven individuals were analyzed and showed that the percent of cells expressing each of these markers increased for stimulated naïve- and MBC-derived B cells with one exception, CD11c, for which the percent of B cells expressing CD11c did not increase significantly in stimulated MBC-derived B cells (Figure 3A). The percent of B cells that expressed each marker was greater for stimulated naïve-derived B cells as compared to MBC-derived B cells (Figure 3A). We also determined the percent of T-bet^+^ B cells that expressed each of the five markers. A high percent of naïve-derived T-bet^+^ B cells coexpressed the three most frequently expressed markers, CXCR3, CD86, and CD95 whereas FcRL5 and CD11c were expressed by a smaller percent of the T-bet^+^ naïve-derived cells (Figures 4A,B, Supplementary Table 2). A similar overall pattern of co-expression was observed for T-bet^+^ MBC-derived B cells (Figures 4A,B, Supplementary Table 2).

For these five markers we also determined if combinations of stimuli in addition to antigen on PLB, CpG, and INFγ were effective in inducing expression. We found considerable heterogeneity in the conditions that induced maximal expression of each of these markers (Figure 3B). For example, for naïve-derived B cells, CD86 was not expressed by B cells immediately *ex vivo* and appeared to be maximally induced by the conditions that induced T-bet expression, namely antigen presented on PLB in the presence of CpG and IFN-γ. In contrast, the induction of CXCR3 expression appeared to be antigen-independent and was highly induced by CpG and IFN-γ alone. The induction of the expression of CD95 also appeared to be relatively antigen-independent. A high percent of naïve B cells expressed FcRL5 *ex vivo* and to a lesser extent CD11c. Culture *in vitro* in the absence of stimulants reduced expression of FcRL5 and CD11c to low levels. However, under a variety of stimulation conditions, the expression of FcRL5 and CD11c was maintained, for FcRL5 near *ex vivo* levels and for CD11c at lower levels, indicating that for FcRL5 and CD11c stimulation *in vitro* promoted maintenance of expression of the markers rather than induction of new expression. The expression of these markers was also verified for FACS sorted naïve and memory B cells cultured under the combination of stimulation conditions and was found to be comparable to that of unsorted cells (Supplementary Table 3).

We also carried out an extensive analysis of the cell surface molecules expressed by naïve- and MBC-derived B cells stimulated with PLB-presented antigen in the presence of CpG and IFN-γ using a BioLegend® human cell screening kit containing antibodies specific for 236 proteins expressed on the surface of human B cells. These data are displayed as log2 of the ratio of the gMFI of stimulated cells over the gMFI of unstimulated cells for tonsil B cells obtained from three individuals (Supplementary Figure 4, Supplementary Table 4). We detected over 200 proteins that were upregulated in stimulated naïve and MBCs relative to the expression on unstimulated B cells including the five atypical MBC markers analyzed individually, CD86, CD95, CD11c, CXCR3, and FcRL5. Transcripts of the genes encoding nearly all of these proteins were detectable in the atypical MBCs from Malian adults with life-long exposure to malaria (Supplementary Table 4). A comparison of transcription of these genes in atypical MBCs and classical MBCs from the same individuals showed that these were differentially regulated suggesting roles for these proteins in the function of atypical MBCs in malaria exposed individuals.

**Figure 5:**
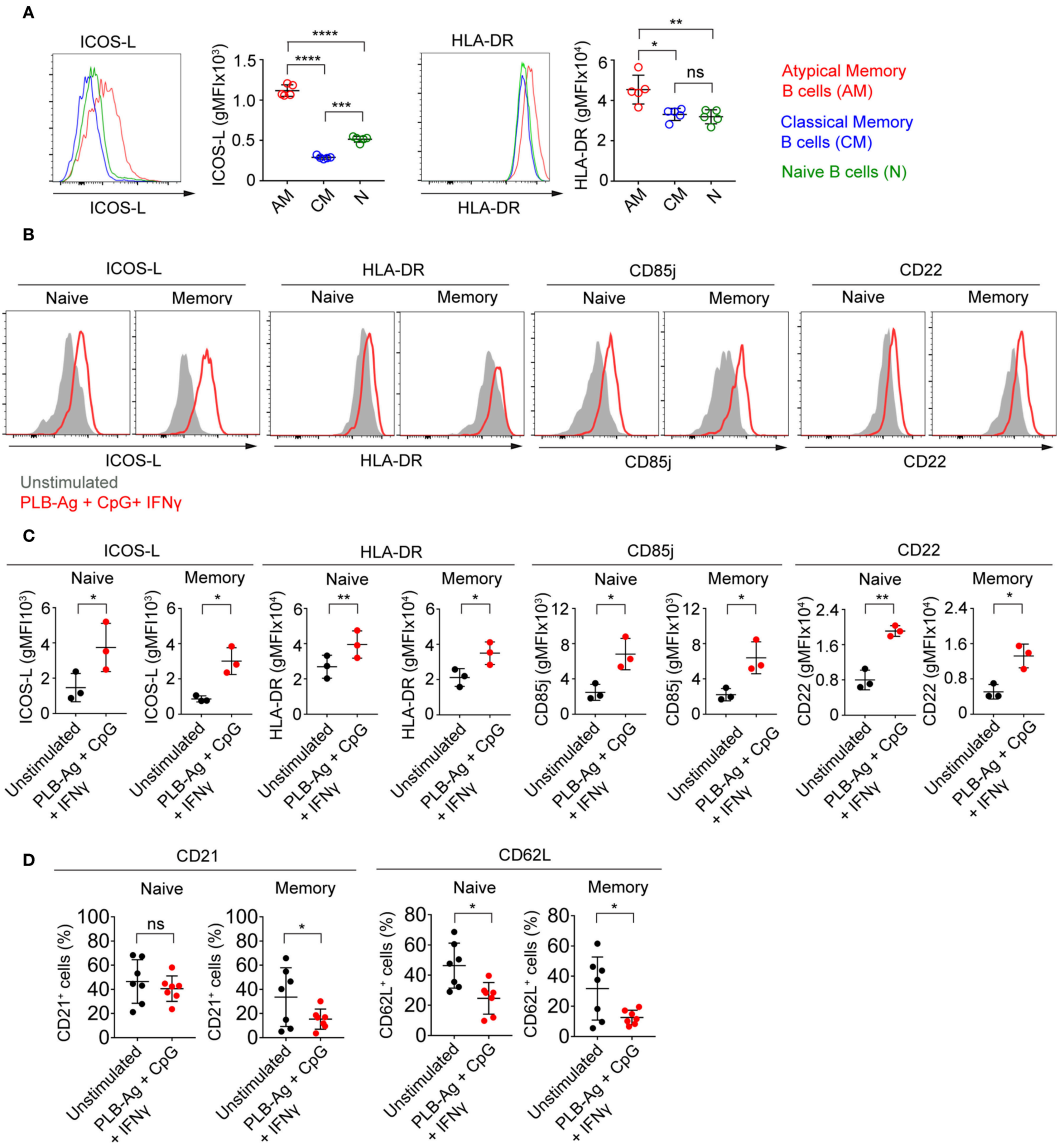
Tonsil naïve and memory B cells stimulated *in vitro* upregulate ICOS-L, HLA-DR, CD85j, and CD22 and downregulate CD21 and CD62L. **(A)** Representative flow cytometry plots of the expression of ICOS-L and HLA-DR by atypical MBCs (CD19^+^ CD21^−^ CD27^−^), classical MBCs (CD19^+^ CD21^+^ CD27^+^), and naïve B cells (CD19^+^ CD21^+^ CD27^−^) in PBMCs isolated from Malian adults with lifelong exposure to malaria. Comparison of expression (gMFI) of ICOS-L and HLA-DR by atypical MBCs, classical MBCs, and naïve B cells (*n* = 5). Data were analyzed using one-way analysis of variance (ANOVA) with Tukey's adjustment. ^*^P < 0.05; ^**^P < 0.01; ^***^P < 0.001; ^****^P < 0.0001; ns, not significant. **(B)** Representative histograms of tonsil naïve and memory B cells following culture *in vitro* for 40 h without stimulation (gray shaded area) or stimulated (red tracing) with antigen presented on PLB in the presence of CpG and IFN-γ and analyzed by flow cytometry for the expression of ICOS-L, HLA-DR, CD85j, and CD22. **(C)** Comparison of the expression (gMFI) of ICOS-L, HLA-DR, CD85j, and CD22 by naïve and memory B cells as in **(B)** (*n* = 3). Data were analyzed using paired *t* test. ^*^P < 0.05; ^**^P < 0.01. **(D)** Percentage of naïve and memory B cells expressing CD21 and CD62L after *in vitro* culture for 40 h either without or with PLB-Ag + CpG + IFN-γ stimulation (*n* = 7). Data was analyzed using paired *t*-test. ^*^P < 0.05; ns, not significant.

The observed increase in expression of CD86, a molecule that plays a critical role in B cell-T cell interactions, led us to ask if two additional molecules involved in B cell-T cell interactions, namely ICOS-L and HLA-DR, were similarly increased in tonsil B cells by culture with antigen presented on PLB in the presence of IFN-γ and CpG. We focused on ICOS-L and HLA-DR as the genes encoding these were previously shown to be differentially expressed in atypical MBCs in malaria exposed African adults as compared to conventional MBCs. We first analyzed by flow cytometry peripheral blood B cells from Malian adults with lifelong exposure to malaria using antibodies specific for CD21 and CD27 to identify naïve B cells (CD19^+^ CD21^+^ CD27^−^), classical MBCs (CD19^+^ CD21^+^ CD27^+^), and atypical MBCs (CD19^+^ CD21^−^ CD27^+^) differentially expressing ICOS-L or HLA-DR. Both ICOS-L and HLA-DR were more highly expressed in atypical MBCs from malaria-exposed individuals as compared to classical MBCs and naïve B cells (Figure 5A). Upon *in vitro* stimulation with antigen on PLB and CpG and IFN-γ, both naïve- and MBC-derived B cells from three tonsil donors showed significantly higher expression of ICOS-L and HLA-DR (Figures 5B,C). The increased expression of ICOS-L and HLA-DR in stimulated as compared to unstimulated cells was also detected using the BioLegend® screening kit (Supplementary Figure 4).

Earlier work also showed that in addition to FcRL5 (a potential inhibitory receptor) the expression of two inhibitory receptors, CD85j and CD22, were upregulated in malaria-associated atypical MBCs as compared to classical MBCs (5). We determined that the expression of both CD85j and CD22 were increased on naïve- and MBC-derived B cells following stimulation with antigen presented on PLB in the presence of CpG and IFN-γ (Figures 5B,C). Increased expression of CD85j and CD22 in stimulated vs. unstimulated B cells was also detected using the BioLegend® screening kit (Supplementary Figure 4).

Previous studies also showed that several markers including CD21 and CD62L are downregulated in atypical MBCs as compared to classical MBCs from malaria-exposed adults. We observed that after stimulation with antigen presented on PLB in the presence of CpG and IFN-γ, both naïve- and MBC-derived T-bet^+^ B cells showed small but significant reductions in the expression of CD62L as compared to unstimulated cells from seven tonsil donors (Figure 5D). The expression of CD21 was reduced on MBC-derived T-bet^+^ B cells but not on naïve B cell-derived T-bet^+^ B cells. Decreases in expression of CD62L was also detected using the BioLegend® screening kit (Supplementary Figure 4).

### Following Stimulation *in vitro* Naïve and MBCs Become Hyporesponsive to Antigen

**Figure 6:**
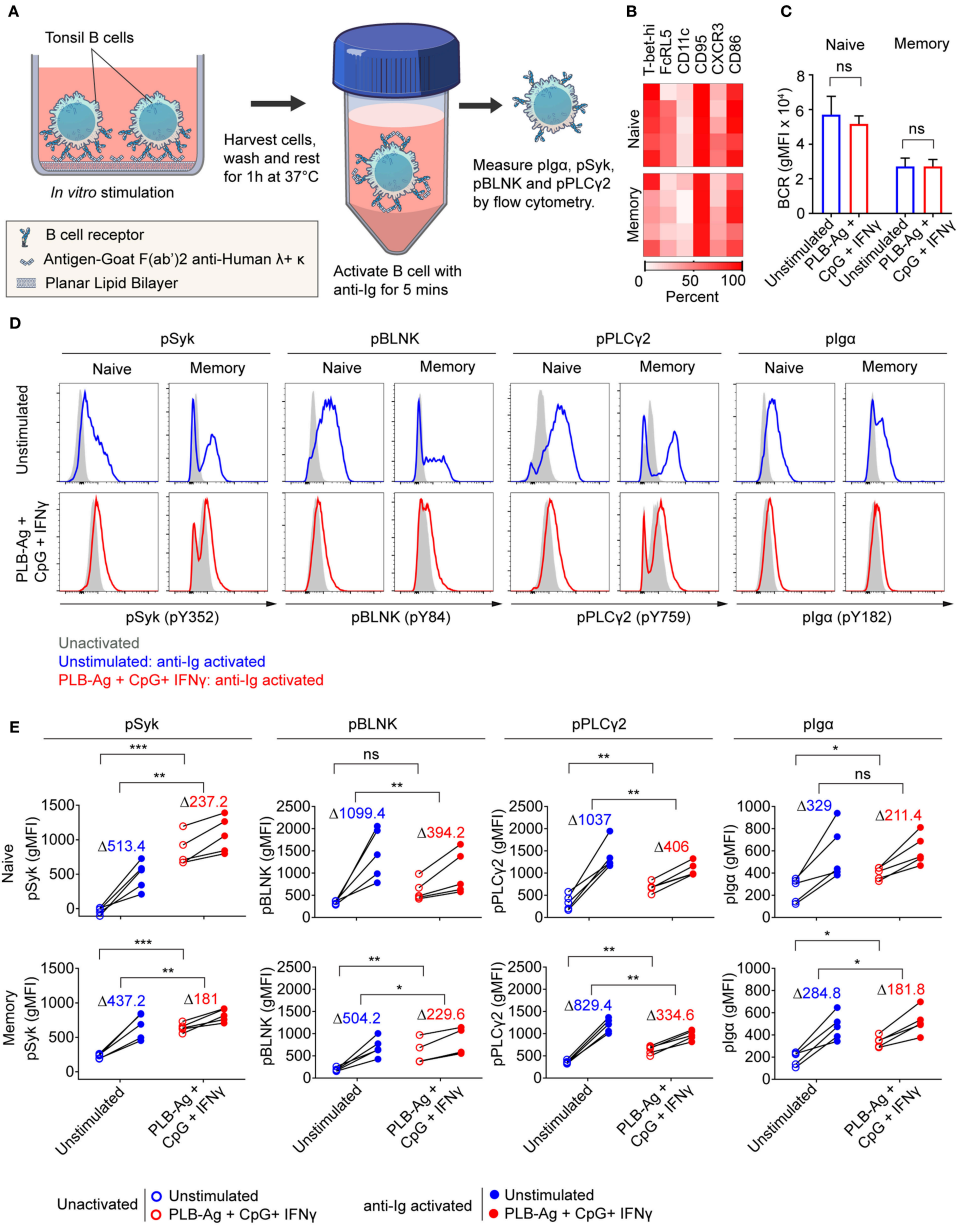
Naïve and memory B cells stimulated *in vitro* to express T-bet exhibit altered BCR signaling. **(A)** Schematic representation of the strategy for measuring the response of tonsil B cells stimulated *in vitro* for 40 h with antigen on PLB in the presence of CpG and INF-γ to subsequent BCR crosslinking. Following *in vitro* culture B cells were harvested, washed and rested for 1 h at 37°C. Cells were activated with soluble F(ab′)_2_ antibodies specific for human IgG + IgA + IgM (H+L) (anti-Ig) for 5 min. Phosphorylation of BCR signaling proteins Syk, BLNK, Igα, and PLCγ2 was analyzed by flow cytometry. **(B)** Heat map indicating the percent of naïve and memory B cells recovered from *in vitro* cultures that expressed T-bet and additional malaria-associated atypical MBC markers from five individuals (rows). **(C)** Comparison of the surface expression of BCR [IgG+IgM+IgA (heavy and light chain)] (gMFI) by naïve and memory B cells recovered from 40 h unstimulated and stimulated cultures (*n* = 3). Data were analyzed using paired *t*-test. ns, not significant. **(D)** Representative histograms indicating expression of phospho-Syk, phospho-BLNK, phospho-PLCγ2, and phospho-Igα in naïve and memory B cells recovered from 40 h cultures *in vitro* and either activated for 5 min with anti-Ig (blue and red tracings) or left unactivated (shaded gray areas). **(E)** Comparison of unactivated (empty circles) and anti-Ig activation (solid circles) induced expression (gMFI) of phospho-Syk, phospho-BLNK, phospho-PLCγ2, and phospho-Igα (*n* = 5) in naïve and memory B cells recovered from 40 h unstimulated and stimulated cultures. Values in the graph indicate the average increase in gMFI induced upon anti-Ig activation [ΔgMFI = gMFI(anti-Ig activated) – gMFI(unactivated)]. Data were analyzed using paired *t*-test. ^*^P < 0.05; ^**^P < 0.01; ^***^P < 0.001; ns, not significant.

We previously described functional differences in BCR signaling between atypical MBCs and classical MBCs from individuals living in malaria endemic areas (2, 6). As compared to classical MBCs, higher basal levels of phosphorylated BCR signaling molecules, most notably Syk and PLCγ2, were observed in malaria-associated atypical MBCs (2, 6) similar to the observation for CD21^low^ B cells from CVID patients (21). Upon BCR stimulation with soluble antigen, the levels of phosphorylated BCR signaling molecules showed a lower fold increase in atypical MBCs as compared to classical MBCs. We tested the capacity of the B cells stimulated *in vitro* to signal through the BCR when engaged with soluble antigen. B cells were cultured without or with stimulation (antigen presented on PLB in the presence of CpG and IFN-γ) for 40 h, harvested, rested for 1 h and then activated with soluble anti-Ig for 5 min (Figure 6A). After stimulation with antigen presented on PLB in the presence of CpG and IFN-γ, both naïve- and MBC-derived B cells expressed the markers associated with atypical MBC as predicted (Figure 6B). Also, expression level of surface BCR was not significantly different in unstimulated and stimulated B cells (Figure 6C). We measured the levels of phosphorylated Syk, Igα, PLCγ2, and BLNK in unstimulated and stimulated B cells after a 1 h rest prior to anti-Ig treatment to establish a base-line level of phosphorylation. Shown are both representative flow plots for the tonsil of one individual (Figure 6D) and the quantification of phosphoprotein levels for those of five donors (Figure 6E). We observed higher basal levels of phosphorylated proteins, particularly phospho-Syk in B cells stimulated *in vitro* as compared to unstimulated B cells (Figures 6D,E). Upon activation with anti-Ig for 5 min the levels of phosphoproteins showed larger increases in unstimulated cells as compared to previously stimulated cells resulting in some cases in equivalent or higher levels of phosphoproteins in unstimulated vs. stimulated cells (Figures 6D,E). Thus, naïve and MBCs stimulated *in vitro* with antigen-presented on PLB in the presence of CpG and IFN-γ to express T-bet and other atypical MBC markers, showed altered BCR signaling capacity, similar to that described for malaria-associated atypical MBCs.

## Discussion

Understanding the cells that serve as a precursor pool for atypical MBCs and the conditions under which atypical MBC differentiation occurs can potentially provide insights for targeted therapies to control the differentiation and function of these B cells in chronic infectious diseases. Our results suggest that both naïve B cells and to a lesser extent, MBCs, in human tonsils have the potential to differentiate into cells that phenotypically and functionally resemble atypical MBCs. Naïve B cells were uniform in their expression of high levels of T-bet upon stimulation with antigen attached to PLBs mimicking presentation by FDCs in the presence of the TLR9 agonist CpG and INF-γ. A smaller fraction of MBCs was induced to differentiate into atypical MBC-like B cells and the level of expression of the phenotypic markers associated with atypical MBCs was less for MBCs as compared to naïve B cells. This heterogeneity of the MBC responses to stimulation *in vitro* may reflect the heterogeneity within the MBC populations defined here simply as IgD^−^, CD10^−^. In contrast, we did not identify conditions under which GC B cells could be induced to express T-bet or other atypical MBC-associated markers. Our previous comparative analysis of several features of atypical MBCs and classical MBCs obtained from malaria-exposed adults including the V_H_ and V_L_ repertoires and number of divisions the cells had undergone suggested that both atypical MBCs and classical MBCs were derived from the same or closely related precursor (2). However, because we know little about the process of proliferative expansion of B cells or somatic mutations or the microenvironments in which these events occur in individuals chronically exposed to malaria these similarities may not be evidence for a common precursor. The data presented here suggests that this precursor may be a naïve B cell or a particular subpopulation of MBCs.

The T-bet^+^ cells generated *in vitro* also expressed a number of markers associated with atypical MBCs in adults living in malaria endemic areas. One issue this finding raises is the role of T-bet in the expression of this array of atypical MBC markers. At present we know very little about the genes in B cells that are under T-bet transcriptional control, so at present the role of T-bet remains an open question. However, our data showed that a variety of the atypical MBC-associated markers are induced independently of T-bet. Moreover, the maximal expression of some markers were in response to stimuli provided *in vitro* that were distinct from those that induce maximal T-bet expression. For example, FcRL5 was maximally induced by antigen presented on PLB alone; maximal CD11c expression resulted from a combination of BCR and TLR9 activation, and CD95 and CXCR3 were maximally expressed by a combination of CpG and IFN-γ. It may be that these proteins function in B cells independently of T-bet expression. It is also possible that the array of proteins induced under conditions that induce T-bet may interact with T-bet to produce a unique intracellular environment that drives atypical MBC generation.

We also provided evidence that the conditions that induce maximal T-bet expression resulted in B cells that appeared to be hypo-responsive to antigen-induced BCR signaling despite comparable surface expression of BCR to unstimulated cells, consistent with our previous observations of atypical MBCs from adults that have life-long exposure to malaria (2, 6). However, the BCR is not inert in T-bet positive cells but rather appears to be maintained in an activated state in terms of protein phosphorylation and fails to response robustly to secondary activation. Atypical MBCs from malaria exposed individuals also fail to secrete cytokines or antibodies under a variety of activation conditions (2). If activation of atypical MBCs fails to induce these basic B cell functions of antibody and cytokine secretion, how might these B cell function? We observed that activation of T-bet expression is accompanied by the expression of a number of markers that play key roles in presentation of antigen to CD4^+^ T cells including HLA-DR, ICOS-L, and CD86. Based on these data we speculate that atypical MBCs may serve to regulate CD4^+^ T cell responses. Recently, acute febrile malaria in African children was correlated with an expansion of Th1-type T follicular helper (Tfh) cells which secrete IFN-γ and are impaired in their helper function (22). It is possible that these Tfh cells contribute to the generation of atypical MBCs and that atypical MBCs in turn regulate these Tfh cells dampening their function with time.

In mice, T-bet^+^, CD11c^+^ B cells termed age-associated B cells (ABC) have been described in aging, autoimmunity, and viral infections (23–25). A variety of conditions have been shown to induce T-bet expression and in some cases CD11c expression in mouse splenic B cells *in vitro* including combinations of stimulation through the BCR, TLR7, or TLR9, in the presence of IFN-γ, IL-21, IL-12, IL-18, or anti-CD40. The ABCs that are expanded in mice during infections with gammaherpesvirus 68, lymphocytic choriomeningitis virus, and vaccinia virus appear to function to produce pathogen specific IgG2a/c antibodies and are involved in clearance of the viruses (17). The ABCs from lupus-prone mice produce autoreactive IgG2a/c antibodies and their appearance is correlated with development of autoimmune disease (25, 26). Thus, in mice T-bet^+^ ABCs appear to function and have either protective or pathogenic role, calling into question the equation of mouse ABCs and malaria associated human atypical MBCs. Recently, it has been reported that in systemic lupus erythematosus patients, T-bet^+^ CD11c^hi^ B cells are expanded and are poised to differentiate into plasma cells (27, 28).

In summary, the results presented here suggest that naïve B cells or subpopulations of classical MBCs may be the progenitors of atypical MBCs in infectious diseases such as malaria in which antigen may be persistently presented in a highly inflammatory environment in the presence of pathogen TLR PAMPs.

## Materials and Methods

### Study Subjects

Fresh human tonsils were obtained from the pathology department of the Children's National Medical Center in Washington, DC following routine tonsillectomies from children. Use of these tonsils for this study was determined to be exempt from review by the NIH Institutional Review Board in accordance with the guidelines issued by the Office of Human Research Protections and were exempted from review. For the study of atypical MBCs from malaria, Malian donors' PBMCs were obtained from individuals enrolled in a cohort study (NIAID protocols 07-I-N141 or 06-I-N147) approved by the ethics committee of the Faculty of Medicine, Pharmacy, and Dentistry at the University of Sciences, Techniques, and Technologies of Bamako, in Mali and reviewed by NIH Institutional Review Board. Informed consent was obtained from all participants. This study was conducted in the rural village of Kalifabougou, Mali where intense *P. falciparum* transmission occurs from June to December each year.

### Isolation of Tonsil B Cells

Tonsils were mechanically disrupted in complete RPMI (RPMI 1640 with L-glutamine supplemented with 10% heat-inactivated FBS, 1 mM sodium pyruvate, 1% MEM nonessential amino acids, 50 μM 2-mercaptoethanol, 100 U/ml penicillin, 100 μg/ml streptomycin, and 25 mM HEPES, pH 7.2–7.5 [all from GIBCO, Invitrogen]) and passed through a 70-μm cell strainer to make a single cell suspension. B cells were then negatively selected using a human B cell enrichment kit (STEMCELL Technologies).

### Preparation of Artificial Antigen-Presenting Membranes

PLB was prepared in 8-well Lab-Tek chamber (#1.0 Borosilicate coverglass system, Nunc) as described before (29). Briefly, PLB was prepared using 110 μM small unilamellar vesicles consisting of 1,2-dioleoyl-sn-glycerol-3-phosphocholine (DOPC) and 1,2-dioleoyl-sn-glycero-3-phosphoethanolamine-N-(cap biotinyl) (DOPE-cap biotin) (Avanti Polar Lipids) in ratio 100:1. To bind Ag to the PLB, the wells containing PLB were incubated at RT with 2.5 μg/ml streptavidin for 10 min, followed by 1 μg/ml biotinylated goat F(ab′)_2_ anti-human λ + κ (Southern biotech) for 20 min.

PMS was prepared in 8-well Lab-Tek chamber (#1.5 Borosilicate coverglass system, Nunc) as previously described (30). Briefly, 293A cells (1 × 10^5^) were seeded in poly-l-lysine-coated wells and cultured overnight in complete RPMI at 37°C, 80–90% confluency being achieved. Cells were washed with HBSS and sonicated with a probe sonicator (5 s, 22% power) in HBSS containing 2% BSA to obtain PMS. To bind Ag to the PMS, the wells containing PMS were first blocked with HBSS containing 2% BSA for 30 min at RT and incubated sequentially for 30 min with 1 μg/ml biotinylated annexin V (Biolegend), 1 μg/ml streptavidin and 0.5 μg/ml biotinylated goat F(ab′)_2_ anti-human λ + κ (Southern biotech).

Ag concentrations for PLB and PMS were selected by titration measurements to contain the same amounts.

### *In vitro* Stimulation of Tonsil B Cells

Tonsil B cells (1 × 10^6^) were stimulated with various combinations of BCR and TLR9 ligand, in presence of cytokines, IFN-γ and IL-12+IL-18. For BCR stimulation, 10 nM biotinylated goat F(ab′)_2_ anti-human λ + κ (Southern biotech) was used for either soluble Ag or membrane-bound Ag on PLB or PMS. B cells were cultured in complete RPMI at 37°C for 40 h in 8-well chambers containing either PLB-Ag or PMS-Ag for membrane Ag engagement and B cells were cultured in round-bottomed 96-well plates for soluble antigen engagement. When needed, cells were supplemented with CpG (ODN2006) (1 μM, Invivogen) in culture media for TLR9 stimulation. For cytokines, rhIFN-γ (50 ng/ml, Biolegend), rhIL-12 (50 ng/ml, Biolegend), and rhIL-18 (50 ng/ml, MBL) were used.

### Flow Cytometry

For analysis of expression of cell surface markers associated with malaria-induced atypical MBCs, *in vitro* cultured cells were harvested, washed in PBS containing 1% BSA and incubated with live/dead fixable stain (Invitrogen) and fluorescently labeled antibodies against CD19, CD10, CD95, CD21, CD27, CD62L from BD Biosciences; CD11c, CXCR3, ICOS-L, HLA-DR, CD85j, CD22 from Biolegend; IgD from Southern Biotech, CD86 from R&D systems and FcRL5 (31). For intracellular T-bet staining, cells were fixed and permeabilized with FoxP3 staining buffer according to the manufacturer's protocol (eBioscience) and stained with antibodies against T-bet from eBioscience. For measuring surface BCR expression levels, cells were incubated with biotinylated F(ab′)_2_ anti-human IgG + IgA + IgM (H+L) (Jackson ImmunoResearch) on ice, followed by fixation with 4% PFA and staining with fluorescently labeled streptavidin. For intracellular phospho-Syk and phospho-BLNK staining, cells were permeabilized with 0.1% Triton for 10 min at RT, followed by overnight staining at 4°C with antibodies against phospho-Syk (pY352) and phospho-BLNK (pY84) from BD Biosciences. For intracellular phospho-Igα and phospho-PLCγ2 staining, cells were permeabilized with methanol on ice for 30 min, followed by overnight staining at 4°C with antibodies against phospho-Igα (pY182) from Cell Signaling technology and phospho-PLCγ2 (pY759) from BD Biosciences. FACS analyses were performed on a BD LSR II flow cytometer (BD Biosciences) and analyzed using FlowJo software (Tree Star, Inc). A detailed information about the antibodies used in this study is given in Supplementary Table 5.

For analysis of cell surface markers using BioLegend® screening kit, purified tonsil B cells (20 × 10^6^) from three individuals were incubated at 37°C for 40 h in 1-well chamber either containing complete RPMI or containing 10 nM biotinylated goat F(ab′)_2_ anti-human λ + κ (Southern biotech) bound to PLB and complete RPMI supplemented with rhIFN-γ (50 ng/ml, Biolegend) and CpG (ODN2006) (1 μM, Invivogen). The cells were harvested and washed in PBS containing 1% BSA and incubated with live/dead fixable stain (Invitrogen) and fluorescently labeled antibodies against IgD from Biolegend and CD10 from BD Biosciences. To differentiate between cells from different individuals and stimulation conditions, the samples were barcoded using antibodies against CD19 from BD Biosciences and CD20 from Biolegend tagged with distinct fluorescent label (32). The cells were then washed, combined and stained for various surface markers using LEGENDScreen™ human cell screening kit (Biolegend). FACS analyses were performed on a BD LSRFORTESSA X-20 flow cytometer (BD Biosciences) and analyzed using FlowJo software (Tree Star, Inc).

### BCR Signaling Assay

Tonsil B cells (1 × 10^6^) were cultured in complete RPMI for 40 h in 8-well chambers containing PLB without any stimulation or with PLB-Ag, rhIFN-γ (50 ng/ml, Biolegend) and CpG (ODN2006) (1 μM, Invivogen). *In vitro* cultured cells were harvested, washed with RPMI 1640 supplemented with 0.1% heat-inactivated FBS and rested for 60 min at 37°C. For surface staining, cells were incubated with anti-IgD Fab fragment (Southern Biotech), anti-CD10 antibody (HI10a) from BD Biosciences and live/dead fixable stain (Invitrogen) for 20 min at RT and washed with RPMI 1640 supplemented with 0.1% heat-inactivated FBS. F(ab′)_2_ anti-human IgG + IgA + IgM (H+L) (Jackson ImmunoResearch) was added to the cells at a final concentration of 10 μg/ml and incubated at 37°C for 5 min, followed by fixation with 4% PFA for 10 min at 37°C. Phospho-proteins of Syk, BLNK, Igα, and PLCγ2 were analyzed by flow cytometry.

### Statistical Analysis

Statistical analyses were carried out using GraphPad Prism 7.04 software and the statistical methods used for each experiment and *P*-value ranges are indicated in figure legend. Data points were assumed to be approximately normally distributed and the data sets were tested using paired *t*-test or repeated-measures one-way ANOVA with Tukey's adjustment.

## Ethics Statement

Fresh human tonsils were obtained from the pathology department of the Children's National Medical Center in Washington, DC following routine tonsillectomies from children. Use of these tonsils for this study was determined to be exempt from review by the NIH Institutional Review Board in accordance with the guidelines issued by the Office of Human Research Protections and were exempted from review. For the study of atypical MBCs from malaria, Malian donors' PBMCs were obtained from individuals enrolled in a cohort study (NIAID protocols 07-I-N141 or 06-I-N147) conducted in the rural village of Kalifabougou, Mali where intense *P. falciparum* transmission occurs from June to December each year.

## Author Contributions

HS, AA, and SP conceived the project, designed the experiments, and edited the manuscript. AA carried out experiments. AA and HS analyzed the data. SN provided non-commercial reagents. AA and SP wrote the manuscript.

### Conflict of Interest Statement

The authors declare that the research was conducted in the absence of any commercial or financial relationships that could be construed as a potential conflict of interest.
