# Supplementary material for: The Differentiation in vitro of Human Tonsil B Cells With the Phenotypic and Functional Characteristics of T-bet+ Atypical Memory B Cells in Malaria
Source: Front Immunol. 2019 Apr 24;10:852. doi: 10.3389/fimmu.2019.00852 (PMC6491666; doi:10.3389/fimmu.2019.00852)
Supplement: Supplementary file 6 [file Data_Sheet_1.docx]

Supplementary Material

**The differentiation *in vitro* of human tonsil B cells with the phenotypic and functional characteristics of T-bet+ atypical memory B cells in malaria**

**Abhijit A. Ambegaonkar^1^, Satoshi Nagata^2^, Susan K. Pierce,^1,3^, and Haewon Sohn^1, 3^**

^1^Laboratory of Immunogenetics, National Institute of Allergy and Infectious Diseases, National Institutes of Health, Rockville, Maryland

^2^Center for Drug Design Research, National Institutes of Biomedical Innovation, Health and Nutrition, Osaka, Japan

^3^SKP and HS are co-corresponding authors:

Susan K. Pierce

NIAID/NIH, 5625 Fishers Lane, Room 4S-04E, MSC 9418, Rockville, MD 20852 USA

E-mail: [spierce@nih.gov](mailto:spierce@nih.gov) Phone: (301) 496-9589 Fax: (301) 594-9990

Haewon Sohn

NIAID/NIH, 5625 Fishers Lane, Room 4S-04D, MSC 9418, Rockville, MD 20852 USA

E-mail: [hwsohn@nih.gov](mailto:hwsohn@nih.gov) Phone: (301) 761-5028 Fax: (301) 594-9990

**1 Supplementary Figures**

**Supplementary Figure 1: Gating strategy for Naïve, Memory and GC B cells**

(A) Gating strategy for tonsil B cells (*ex vivo* and after *in vitro* culture for 40 h without or with PLB-Ag + CpG + IFN-γ stimulation). B cell subsets were gated based on IgD and CD10 expression into Naïve (CD10^-^, IgD^+^), Memory (CD10^-^IgD^-^) and GC (CD10^+^IgD^-^) B cells. Viability (percent live cells) of total B cells cultured for 40 h without (black dots) or with PLB-Ag + CpG + IFN-γ stimulation (red dots) is shown in the graph alongside.

(B) Strategy for FACS sorting of tonsil B cells into Naïve, Memory and GC B cells. Tonsil B cells were stained using fluorescently labelled anti-IgD Fab fragment and anti-CD10 antibody and FACS sorted into Naïve (CD10^-^, IgD^+^), Memory (CD10^-^IgD^-^) and GC (CD10^+^IgD^-^) B cells. Sorted cells were cultured *in vitro* for 40 h without or with various stimulations. Viability (percent live cells) of FACS sorted Naïve (orange dots), Memory (purple dots) and GC (green dots) B cells cultured for 40 h without or with PLB-Ag + CpG + IFN-γ stimulation is shown in the graph alongside.

(C) Comparison of T-bet expression (gMFI) by Naïve B cells stimulated *in vitro*. Tonsil B cells were cultured *in vitro* for 40 h on PLB with combination of Ag, CpG or IFN-γ. T-bet expression in Naïve (CD10-, IgD+) B cells was analyzed by Flow cytometry. Data were analyzed using one-way analysis of variance (ANOVA) with Tukey’s adjustment. *, P < 0.05; ***, P < 0.001; ****, P < 0.0001; ns, not significant.

**Supplementary Figure 2: T-bet expression in tonsil B cells upon *in vitro* stimulation**

Tonsil B cells were cultured *in vitro* for 40 h on PLB with combination of Ag, CpG, IFN-γ or IL12+18. T-bet expression in Naïve (CD10^-^, IgD^+^), Memory (CD10^-^IgD^-^) and GC (CD10^+^IgD^-^) B cells was analyzed by Flow cytometry. Shown are representative histograms indicating T-bet expression in stimulated cells (red curve) or in unstimulated cells (solid gray curve). The stimulation conditions are as indicated in red and are grouped into single stimulus, double stimuli and triple/quadruple stimuli.

**Supplementary Figure 3: T-bet expression in unsorted and FACS sorted Naïve and Memory B cells upon *in vitro* stimulation**

Total tonsil B cells or FACS sorted Naïve (CD10^-^, IgD^+^) and Memory (CD10^-^IgD^-^) were cultured *in vitro* for 40 h on PLB with combination of Ag, CpG, IFN-γ or IL12+18. T-bet expression was analyzed by Flow cytometry. The graph indicates the average gMFI of T-bet expression in Naïve and Memory B cells after *in vitro* stimulation under the conditions indicated.

**Supplementary Figure 4: The effect of *in vitro* stimulation on expression of B cell surface proteins**

Tonsil B cells were cultured *in vitro* for 40 h without or with PLB-Ag + CpG + IFN-γ stimulation. Surface expression of various proteins expressed by Naïve and Memory B cells (listed in Table S2) was assessed by flow cytometry. The graph indicates rank order expression of the surface proteins in Naïve and Memory B cells after *in vitro* stimulation. Each blue dot indicates fold change in gMFI of protein expression by PLB-Ag + CpG + IFN-γ stimulated cells relative to unstimulated cells (log_2_ scale) (n=3). Atypical MBC markers are highlighted as red dots. Black line indicates the mean value.

**2 Supplementary Tables**

**Supplementary Table 1: T-bet expression by unsorted and FACS sorted Naïve, Memory and GC B cells stimulated *in vitro***

Tonsil B cells were cultured *in vitro* for 40 h with combination of antigen, either soluble or presented on a planar lipid bilayer (PLB) or plasma membrane sheet (PMS), CpG, IFN-γ or IL-12 + IL-18. T-bet expression in Naïve (IgD^+^CD10^-^), Memory (IgD^-^CD10^-^) and GC (IgD^-^CD10^+^) B cells was analyzed by Flow cytometry. The values in the table indicate T-bet gMFI (n = 3) calculated for conditions in which a minimum of 5% of the cells were T-bet^+^.

**Supplementary Table 2: Expression of malaria-associated atypical MBC markers by T-bet^+^ Naïve and Memory B cells after *in vitro* stimulation**

Tonsil B cells were cultured *in vitro* for 40 h with combination of antigen, either soluble or presented on a planar lipid bilayer (PLB) or plasma membrane sheet (PMS), CpG, IFN-γ or IL-12 + IL-18. Expression of T-bet, FcRL5, CD11c, CD95, CXCR3 and CD86 was determined by flow cytometry for Naive (IgD^+^CD10^-^) and Memory (IgD^-^CD10^-^) B cells. The values in the table indicate the percent of B cells positive for T-bet and other surface markers (n = 3).

**Supplementary Table 3: Expression of malaria-associated atypical MBC markers by FACS sorted Naïve and Memory B cells after *in vitro* stimulation**

Tonsil B cells were FACS sorted based on IgD and CD10 expression into Naïve (CD10-IgD+), Memory (CD10-IgD-) and GC (CD10+IgD-) B cells and cultured *in vitro* with either soluble antigen or antigen presented on PLB in the presence of the stimuli shown. Expression of FcRL5, CD11c, CD95, CXCR3 and CD86 was determined by flow cytometry. The values in the table indicate the percent of B cells expressing each marker (n = 3).

**Supplementary Table 4: Differential expression of various cell surface proteins in naïve and memory B cells after *in vitro* stimulation.**

Tonsil B cells were cultured *in vitro* for 40 h without or with PLB-Ag + CpG + IFN-γ stimulation. The cells were stained for various surface markers using LEGENDScreen^TM^ human cell screening kit (Biolegend) and analyzed by flow cytometry. The data are displayed as log2 of the ratio of the gMFI of stimulated cells over the gMFI of unstimulated cells for tonsil B cells (n = 3). Relative difference in transcripts of these surface markers for atypical MBCs and classical MBCs from Malian adults with life-long exposure to malaria is also included in this table.

**Supplementary Table 5: List of antibodies**

Information about fluorescently labelled antibodies used for analysis of expression of cell surface markers and intracellular proteins is given, along with the concentration or the dilution factor for each antibody.
