# Supplementary figures and images for: The Differentiation in vitro of Human Tonsil B Cells With the Phenotypic and Functional Characteristics of T-bet+ Atypical Memory B Cells in Malaria
Source: Front Immunol. 2019 Apr 24;10:852. doi: 10.3389/fimmu.2019.00852 (PMC6491666; doi:10.3389/fimmu.2019.00852)

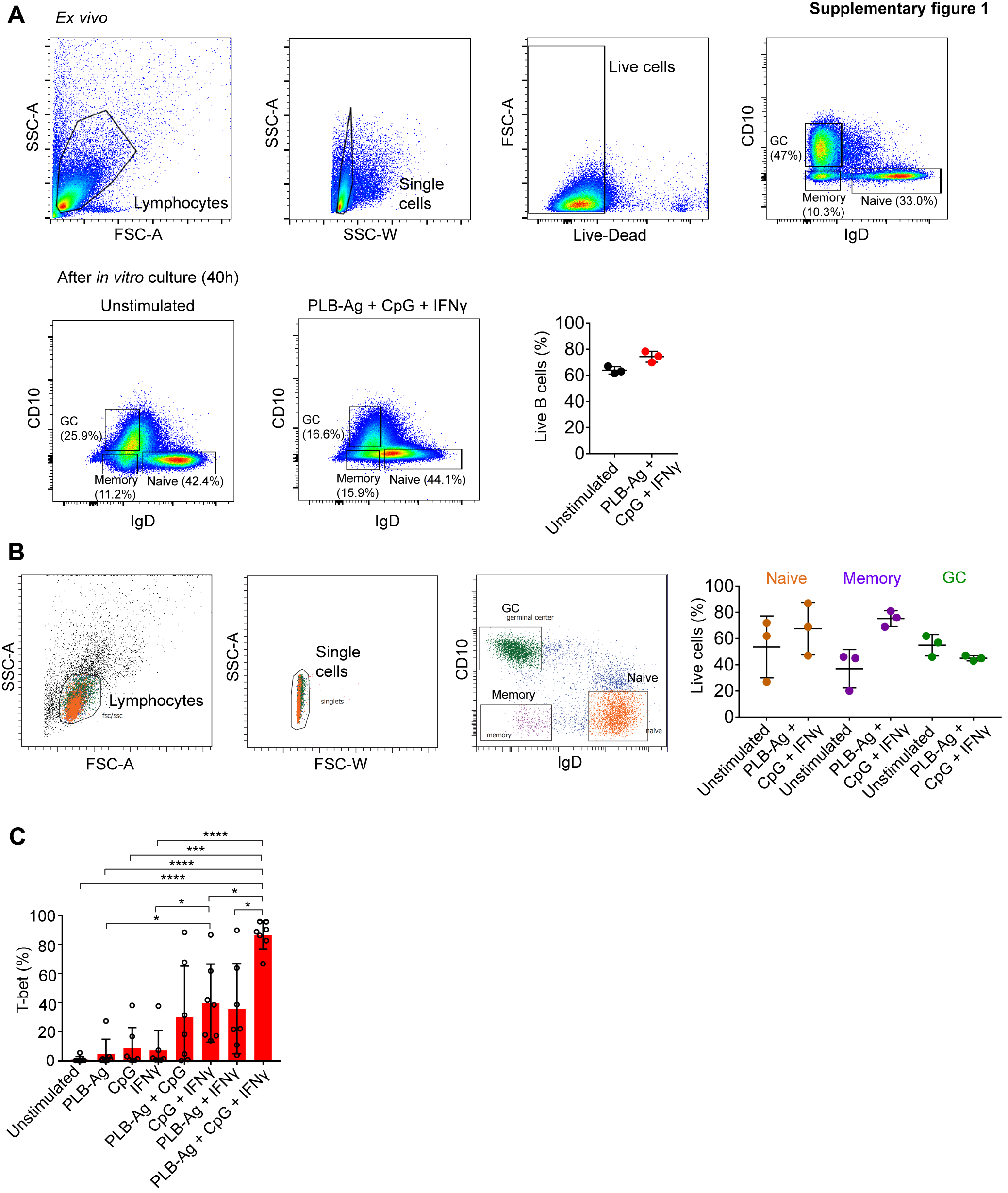

Supplement: Supplementary file 7 [file Image_1.TIF]

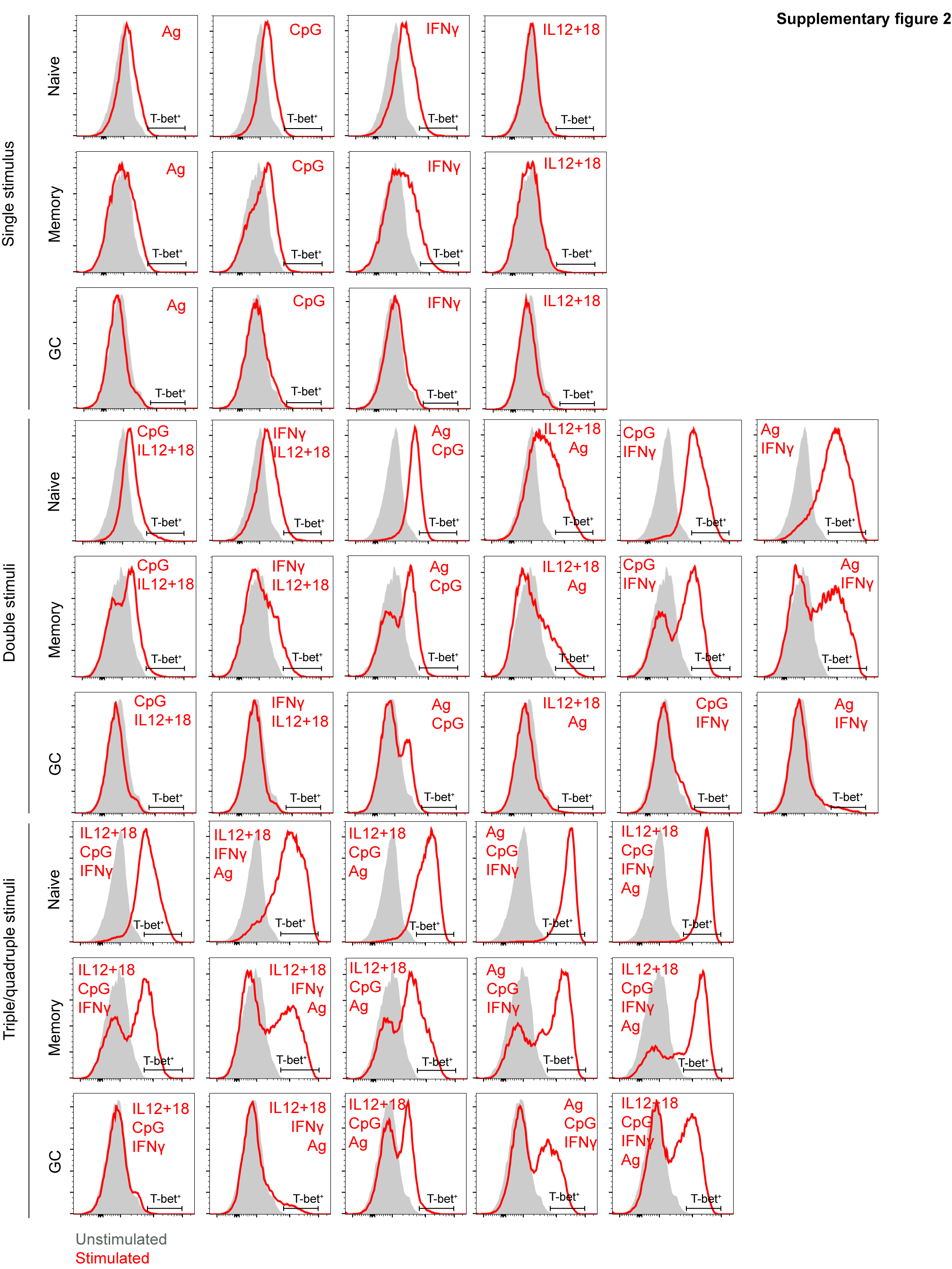

Supplement: Supplementary file 8 [file Image_2.TIF]

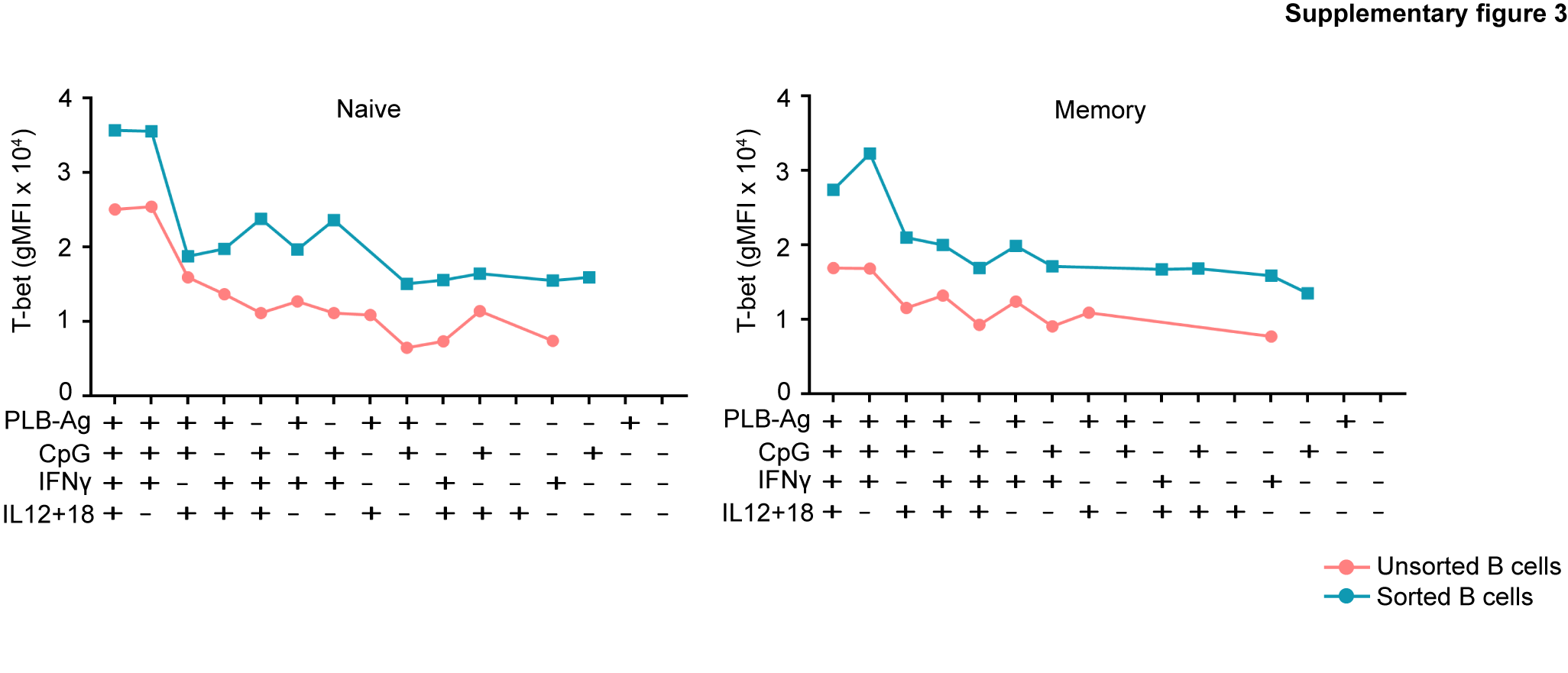

Supplement: Supplementary file 9 [file Image_3.TIF]

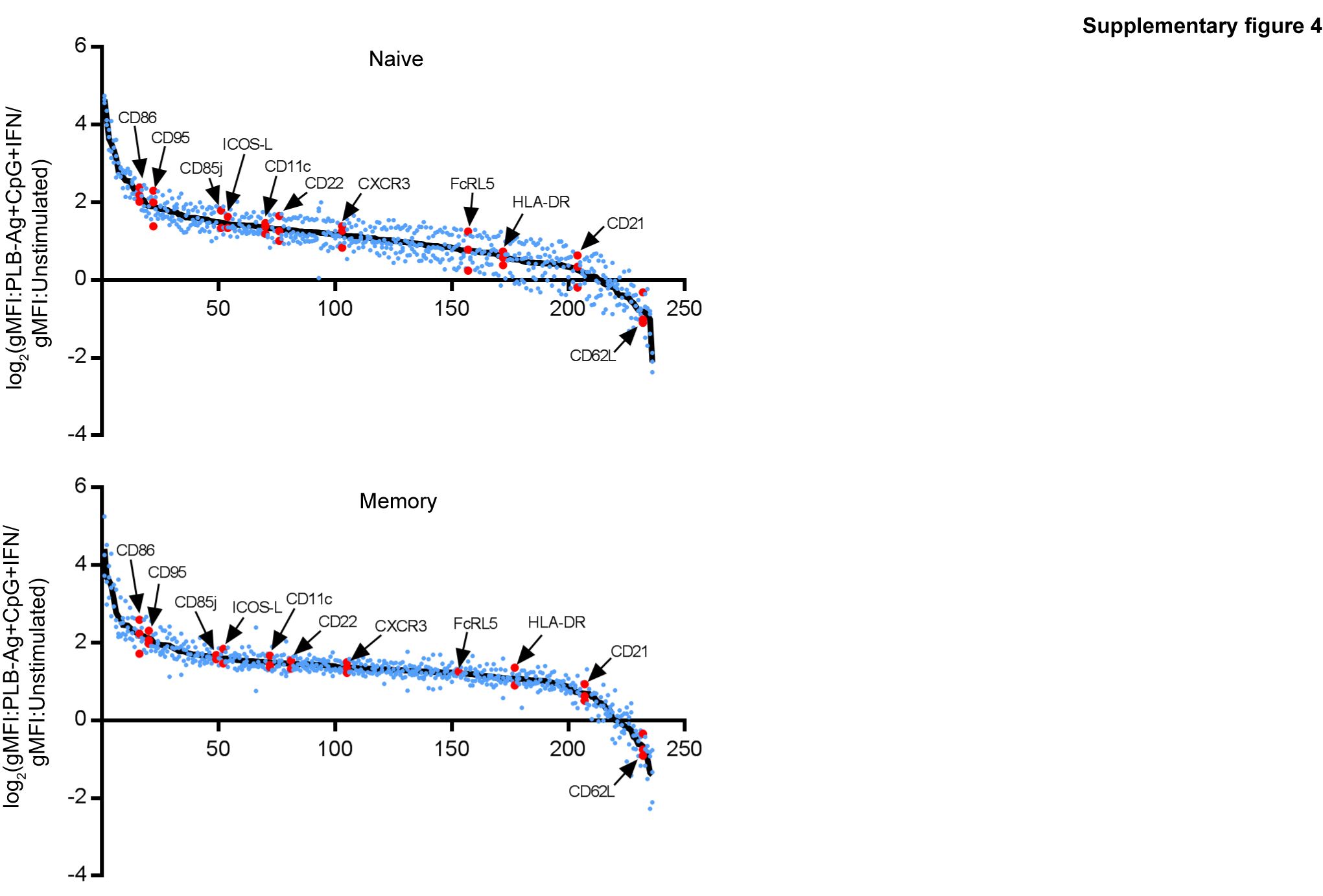

Supplement: Supplementary file 10 [file Image_4.tif]
